# Begin the Day Aright

**Published:** 1892-09

**Authors:** 


### BEGIN THE DAY ARIGHT.

A good beginning is always desirable in a day of work or pleasure. A few cheerful words count for more now than at any other time, for they often serve as a keynote for the whole day. It depends largely upon the mother of a family whether home is a sunny resting place or merely a habitation of complaint and contention. Unhappy indeed is the household that begins the morning with domestic clouds. There are some heads of families who seem to consider it due to their dignity that they should perpetually wear a severe aspect and who are never sterner or more unrelenting than at the breakfast table. The family leave for their respective daily tasks with a sensation of chilliness that requires the most cheerful surroundings to overcome.

There are mothers who begin the new day with recounting all the minor vexations of the day before. The husband and sons who are hurrying off to business are compelled to listen to the grievances with servants and other petty afflictions which the mistress may choose to rehearse. It is hardly strange if they inwardly wonder if all the tales of household woes are not due to bad management, so continual is the problem presented. The thoughtful mother airs none of her domestic trials at the breakfast table. Here is an atmosphere of serenity and sunshine.

A sunny word now goes far to lighten the day's tasks, to speed the parting members of the family and to help those who remain behind in the performance of their various duties. The hostess who goes to her kitchen and deftly straightens out with a few touches of her own the tangled skein of work which she may find there, accomplishes more by a few well chosen words of encouragement than she will by a score of complaints. Consideration and kindness often do wonders with even the most stupid and obdurate of servants. It shows great selfishness for the family or for the mother to make other members of the household bear the burden of their individual trials and grievances at the breakfast table. Each one has a right to a cheerful beginning of his day's work.

